# “Before I came to the hospice, I had nobody”. A qualitative exploration of what patients, family-caregivers, clinicians and volunteers valued most about home, day therapy or inpatient hospice services

**DOI:** 10.1177/26323524241231820

**Published:** 2024-02-28

**Authors:** Nicole Marie Hughes, Jane Noyes, Carys Stringer, Trystan Pritchard

**Affiliations:** School of Medical and Health Sciences, Bangor University, Bangor, UK; School of Medical and Health Sciences, Bangor University, Bangor LL57 2DG, UK; Centre for Health Economics and Medicines Evaluation, Bangor University, Bangor, UK; St David’s Hospice, Llandudno, UK

**Keywords:** caregiver, day therapy, end-of-life care, healthcare professionals, hospice, hospice at home, inpatient, palliative care, qualitative, value

## Abstract

**Background:**

Globally, the demand for hospice care continues to grow resulting in substantial resource burden. Whilst some countries are able to rely on fixed government contributions, statutory funding for palliative care in the United Kingdom is unequally distributed. These unstable funding streams and increased demand means that hospices need to evidence their value.

**Objective:**

This study explored the experiences of patients and family-caregivers to determine what they valued most from accessing hospice services in Wales.

**Methods:**

In this large multi-site qualitative study, 94 semi-structured interviews and 2 focus groups were conducted with hospice patients (*n* = 45), family-caregivers (*n* = 18), hospice staff (*n* = 31) and volunteers (*n* = 10). The audio recordings were transcribed verbatim and analysed using Framework analysis.

**Results:**

Seven themes described patient and family-caregiver experiences and what they valued most: relationships with staff and volunteers, greater support networks which reduced social isolation and loneliness, provision of information and advice which improved patient autonomy, symptom management and subsequent reduction in psychological distress, improvements in patient functionality, mobility and overall physical health and respite relief which promoted improved relationships.

**Conclusion:**

This is the largest study to explore what patients and family-caregivers value from hospice care. Findings indicate that hospice care provides a truly needs-led and strengths-based service to those who are nearing and at the end-of-life, which is highly valued by patients and family members.

## Background

Palliative and hospice care are both facets of end-of-life care, that can be provided within a dedicated hospice site or at home, and provides symptom management, comfort and support to both people with a terminal condition and their families. Palliative and hospice care is increasingly recognized globally as a human right with growing evidence of cost effectiveness.^1^ It is estimated, however, that just 14% of people who require palliative care will receive it.^2^ An international ranking exercise assessed the United Kingdom (UK) as a country that provides the best quality of death in the world,^3^ a finding which was echoed by Finkelstein *et al.*^4^ Despite this, there are substantial differences in the way that hospice care across the UK is funded when compared with other countries. Whilst the UK relies on a proportion of statutory funding ranging from 20% to 50%^5^ which is distributed unequally across hospice facilities, New Zealand which ranked third on the international ranking exercise,^3^ receives a fixed proportion of government contribution (70%).^1^ As demand for palliative care increases due to an ageing population, the current funding model is unsustainable despite hospice care providing a resource efficient alternative to the cost of acute hospital care.^6^

Hospice funding is generally uncertain^1^ and with the current cost of living crisis expected to push hospices into a deficit of £186 million,^7^ evidencing the value of hospice care is vital for their future sustainability. Funding pressures will be further exasperated by increased demand as it is estimated that between 25% and 47% more people will require palliative care by 2040 in England and Wales.^8^ Adult hospice services provide vital palliative care services to patients with a terminal diagnosis and includes a care quotient for families and caregivers, however, there is a need for services to further evolve to better accommodate anyone with palliative and end-of-life care needs and not just people dying with cancer.^9^ The evolution of services must be based on good quality evidence and include service user perspectives, but it remains challenging to engage with people who are dying for the sole purpose of research.^10^ Consequently, much of the debate surrounding service user perspectives, particularly the patient experience, has often been defined by and filtered through the views of others or through the inclusion of patients who are not considered to be nearing death – typically those utilizing day therapy or respite services. Subsequently, less is known about people with poorer quality of life. Failing to engage directly with and understand the views, experiences and values of all patients, irrespective of age, diagnosis, socio-economic background and ethnicity will inevitably have implications for future healthcare delivery as services fail to adapt to individual patient need.^11^ Whilst indicators of quality are often determined using satisfaction questionnaires, the responses to these often lack the richness of data that can be acquired through the active involvement of stakeholders in qualitative research, and thus may not be sufficiently grounded in the values of stakeholders when used in isolation.^11^ A mixed-methods review conducted by the authors identified the need for a primary qualitative study.^12^ Despite well-documented ethical concerns,^13^ additional qualitative research with palliative populations is required to explore experiences and values, which are often complex and multi-faceted. The aim of this qualitative study was to address this research gap by exploring what patients and family-caregivers valued most from hospice care that could contribute to the future development of effective services for all palliative care patients, irrespective of diagnosis. Patient and family-caregiver views and experiences were supplemented by those of hospice healthcare professionals and volunteers.

## Methods

### Study design

A qualitative design informed by the five-stage framework method of data collection and analysis as described by Ritchie *et al.*^14^ was used because of its suitability for applied policy research. This approach fits well with a critical realist epistemological position. The five stages are described in detail in the data analysis section below.

### Participant recruitment

Two strategies were employed to facilitate recruitment: (1) the facilitation of regular meetings with the hospice personnel (staff and volunteers) to promote the study and (2) the appointment of a designated member of staff who was responsible for the dissemination of recruitment material to the appropriate person(s). Participant facing materials were developed in collaboration with the North West Wales Cancer Forum.

Hospice stakeholders which included patients, family-caregivers, hospice staff and volunteers (Table 1) who had direct or indirect experiences of one or more of the following hospice services were purposively or conveniently recruited from the: (1) inpatient unit, (2) day therapy service or (3) at home service in either a personal or a professional capacity. Sampling and data collection continued until data saturation. The inclusion criteria for each stakeholder differed (Table 2). Staff and volunteers had to be a current employee while for patients and family-caregivers, they needed to have experience of one or more of the aforementioned hospice services. Patient participation was not dependent on the enrolment of their family-caregiver and for patients deemed unable to provide informed consent, a personal consultee was sought. Due to resource restriction, participants who were unable to communicate through the medium of English or Welsh were excluded. Four hospice sites were included in this study.

**Table 1.** Sampling framework.

Recruitment phase	Hospice stakeholder	Method	Rationale
Phase I	Clinical and non-clinical personnel	1-2-1 interview	A range of clinical and non-clinical roles were purposively sampled by profession (e.g. doctor, nurse, chef) to explore their perspectives on working within a palliative care setting and the perceived effect of their role on patients and family-caregivers. All hospice sites contributed to this dataset.
	Volunteers	Focus group	
Phase II	Patients	1-2-1 interview or interviews as patient–family-caregiver dyad	Through convenience sampling – a non-probabilistic sampling approach, patients and family-caregivers were invited to interview to discuss their personal experiences of hospice care. Recruitment continued until data saturation. While this resulted in the under-representation of both male patients and patients with non-malignant diseases, this is representative of the breakdown at each site.
	Family-caregivers	1-2-1 interview or interviews as a patient–family-caregiver dyad	

**Table 2.** Inclusion/exclusion criteria.

Stakeholder	Inclusion criteria	Exclusion criteria
Patients	Experience: Direct experience of hospice care at one or more of the included hospice sites. Language: English or Welsh Age: >18	Experience: Direct or indirect experience at a hospice site which was not included in the study. Language: Any language other than English or Welsh Age: <18
Family-caregivers	Experience: Direct or indirect experience of hospice care at one or more of the included hospice sites. Language: English or Welsh Age: >18	Experience: Direct or indirect experience at a hospice site which was not included in the study. Language: Any language other than English or Welsh. Age: <18
Hospice staff/volunteers	Experience: Current employee or volunteer at one or more of the hospice sites. Language: English or Welsh Age: >18	Experience: Previous employee or volunteer at one or more of the hospice sites. Language: Any language other than English or Welsh. Age: <18

A purposive sample of paid and volunteer personnel to report as proxies on what patient and family-caregivers valued were recruited as they may offer an important proxy perspective on the value of hospice care (Table 1). A convenience sample of patients and family-caregivers from each hospice site was recruited. A researcher (NMH) was embedded at sites to build rapport with potential participants and enable swift recruitment of study participants. Following receipt of a ‘consent to contact’ form distributed by hospice personnel, a mutually convenient time and location for data collection was arranged.

### Data collection

To elicit the views and experiences of stakeholders in sufficient depth, a conversational approach to qualitative data collection was adopted alongside the use of a semi-structured topic guide. Following advice from the hospice Chief Executive Officers, to encourage recruitment, volunteers were invited to join a focus group while staff, patients and family-caregivers were invited to participate in a semi-structured interview. The first couple of interviews from each of the separate stakeholder groups were used to pilot the topic guide and any necessary changes were made. Data collected during the piloting phase was retained. Multiple perspectives were sought on what patients and their family members valued from hospice care as it was not appropriate for some patients and their families to participate in a research study due to their clinical condition and proximity to end-of-life. We felt that proxy perspectives from hospice volunteers and staff would add an additional dimension and help fill in the gaps for those patients and their family members who could not participate. We appreciate that proxy perspectives and accounts are not the same as patient and family member lived experiences, but nonetheless these accounts are drawn from people in the same context who are in close proximity for long periods of time when caring for people receiving hospice care. Their interviews were based on their own lived experience of caring and what they thought that patients and family members valued. In reporting these data, we make clear the participant perspective and where accounts are similar or different. Subsequently, priority was not given to one dataset.

### Data analysis

**Figure 1.**
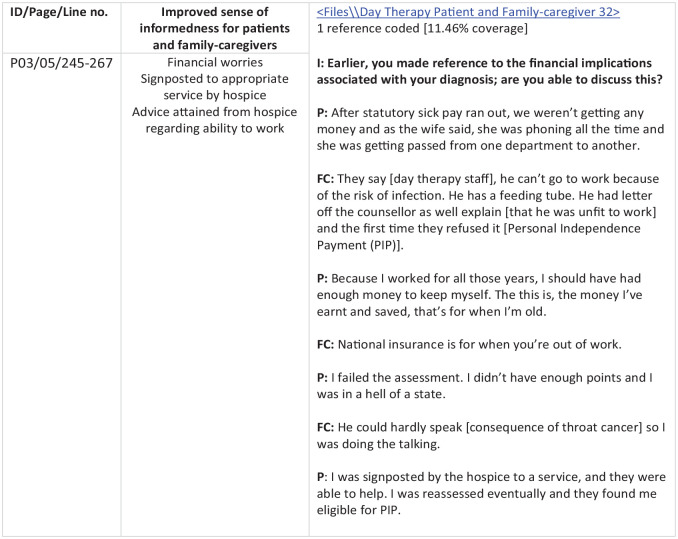
An extract from NVivo demonstrating the charting process used for one theme. FC, family-caregiver; I, interviewer; P, patient.

In the first stage of Framework analysis – *Familiarization*, 26 interviews and 2 focus groups were transcribed verbatim by NMH and line-by-line coded. In stage 2 – *Developing a Theoretical Framework –* the initial *a priori* coding framework developed from a previous systematic literature review conducted by the authors^12^ was used with NVivo. Additional inductive coding ensured that new experiences and perspectives of participants were captured. Through deductive and inductive coding, there were several iterations of the thematic framework, which ensured a comprehensive data-driven approach. Next, during the *indexing* stage, the framework of codes was systematically applied to each transcript revising or merging themes as required. In stage 4 – *Charting* (Figure 1) – data were coded with reference to the thematic framework, and a matrix was designed to manage and summarize these data by theme. In the final stage – *Mapping and interpretation*; the charted data was reviewed and compared against its original form and themes merged, split or renamed as required. The Consolidated Criteria for Qualitative Research reporting guidelines^15^ guided reporting.

### Ethical considerations

The study was highly ethically sensitive. Recruitment of patients was guided by hospice personnel who approached those with capability to talk with a researcher. A distress protocol was used with participants and the researcher who collected data was debriefed and supported. Information on additional support available was signposted to patients and caregivers. Written informed consent was obtained from participants. Patients/family members were reassured that participation or not would not affect any aspect of their care.

### Reflexivity

Team members were predominantly female and represented various perspectives (nursing, public health, methodological, community volunteering) with different levels of prior exposure to palliative care (extensive exposure to limited exposure). Key stakeholders provided additional input from other perspectives (e.g. hospice management, hospice nursing and volunteering) and added gender balance to the all-female team. Regular research team meetings were held to make transparent potential biases and to discuss data collection methods, the coding process, analysis and interpretation of emerging findings. Findings were shared with key stakeholders for feedback.

### Patient and public contribution

Participant facing materials in addition to the topic guides were developed in collaboration with the North West Wales Cancer Patient Forum. At different stages, the involvement of patient and public contributors acted as a catalyst to finding practical solutions to any barriers to completing the study.

## Findings

Ninety-six participants were recruited from four hospice services (Tables 3 and 4). Notably, patients accessing day therapy were typically less unwell at the point of referral for potential participation in the study than those who required inpatient support, which is the likely cause of the imbalance in recruitment.

**Table 3.** Participant characteristics of patients and family-caregivers.

Participant characteristics	Inpatient unit		Day therapy unit	
	Patients (*n* = 10)	Family-caregivers (*n* = 4)	Patients (*n* = 35)	Family-caregivers (*n* = 14)
Sex				
Female	7 (70)	3 (75)	20 (57)	8 (57)
Male	3 (30)	1 (25)	15 (43)	6 (43)
Age (years)				
25–34	–	1 (25)	–	–
35–44	–	–	–	1 (7)
45–54	–	2 (50)	2 (6)	2 (14)
55–64	–	–	7 (20)	6 (43)
65–74	6 (60)	–	10 (29)	3 (21)
75–84	3 (30)	–	10 (29)	2 (14)
85+	1 (10)	1 (25)	6 (17)	–
Diagnosis				
Cancer	7 (70)	N/A	22 (63)	N/A
Non-cancer	3 (30)	N/A	13 (37)	N/A
Ethnicity				
White/White British	10 (100)	4 (100)	35 (100)	14 (100)

Total participants, *N* = 63.

**Table 4.** Participant characteristics of hospice personnel.

Professional category	Role	Inpatient unit	Day therapy unit	Home service	Total, *n* = 33 (%)
Healthcare professionals	Nurse	X^a^	X	YZ	03 (09)
	Senior specialty doctor	X	X	–	01 (03)
	Health support worker	X	XY	Z	03 (09)
	Advanced nurse practitioner	XY	XY	–	02 (06)
	Consultant	X	–	–	01 (03)
	Matron	X	X	–	01 (03)
Social care professionals	Social worker	X	X	–	01 (03)
	Day therapy lead	–	X	–	01 (03)
Therapists	Physiotherapist	XY	XY	–	02 (06)
	Occupational therapist	X	X	–	01 (03)
	Music therapist	X	X	–	01 (03)
	Complementary therapist	XY	XY	–	02 (06)
	Diversional therapist	X	X	–	01 (03)
Volunteers	Volunteers	Q R S T U V W X Y Z		–	10 (30)^b^
Other personnel	Chef	X	X	–	01 (03)
	Community fundraiser	X	X	–	01 (03)
	Reverend	X	X	–	01 (03)

Total participants, *n* = 41; total interviews, *n* = 31; total focus groups, *n* = 2.

a Different letters indicate different staff members whereas the same letter indicates that the staff member worked across one or more of the units (e.g. the second row reveals that just one senior specialty doctor was recruited but worked across both the inpatient and the day therapy unit).

b Ten participants participated in two focus groups. The first focus group consisted of four participants while the second had six participants.

In total, 45 h of interviews were conducted, 30 h of which were conducted with patient and family-caregivers while 15 h were conducted with staff. The interviews lasted between 11 and 105 min. In addition, two focus groups were conducted which lasted 65 and 99 min, respectively. Although the final purposive sample of hospice staff and volunteers was predominantly female, this imbalance is reflective of the gender balance present at each of the study sites at the time this study was conducted.

Table 5 summarizes the many aspects of care that patients and family-caregivers valued across each service. Seven themes were derived from the data which also revealed similarities between what patients and family-caregivers identified as valuable aspects of care. Although staff and volunteers were largely able to identify what patients and family-caregivers valued, they failed to identify themselves as contributors to the provision of an excellent and much valued service.

**Table 5.** A summary of values across stakeholder groups.

What do patient and family-caregivers value about hospice services?	Inpatient				Day therapy			
	Patient	Family-caregiver	*Paid personnel	*Volunteer	Patient	Family-caregiver	*Paid personnel	*Volunteer
The formation of relationships with staff and volunteers								
The personalities, expertise and specialized skills of hospice personnel	✗	•			✗	•		
Timely access to a wide range of staff, services and activities	✗	•			✗	•		
Time spent with staff	✗		✗ •	✗ •	✗		✗ •	✗ •
The availability of hospice volunteers provided additional company			✗ •	✗			✗ •	✗
Greater support network reduced social isolation and loneliness								
The provision of social opportunities					✗		✗	
Opportunities to develop meaningful relationships					✗			
Access to an onsite café		•		•				•
Provision of information and advice improved patient autonomy								
The sense of control and autonomy	✗		✗		✗		✗	
Being prepared for death	✗		✗ •				✗ •	
Availability and accessibility of the hospice services	✗	•	✗ •	✗ •	✗	•	✗ •	✗ •
Access to practical support including financial and domestic support and signposting to other agencies			•		✗	•	✗ •	
The provision of clinical information and advice	✗	•	✗ •		✗	•	✗ •	
Symptom management reduced psychological distress								
Support to maintain psychological, spiritual and emotional well-being	✗			✗ •	✗		✗	✗ •
Symptom management	✗		✗		✗		✗ •	
Improvements in patient functionality and mobility								
Improved mobility	✗				✗			
Improved independence	✗				✗			
Respite relief contributed towards improved relationships								
Respite care to allow valued breaks for family-caregivers	✗	•	•			•	•	
Physical, practical and psychological support for family-caregivers	✗ •	✗ •	✗ •	✗ •	✗ •	✗ •	✗ •	✗ •
Improvements in overall physical health								
Access to personalized catering	✗		✗	✗	✗		✗	✗
Access to a range of complementary therapies	✗		✗	✗	✗	✗	✗	✗

• , Family-caregiver; ✗, Patient.

* Proxy views (hospice personnel) of what patients and family-caregivers value.

## The formation of relationships with staff and volunteers

### Patient and family perspectives

The presence of highly qualified staff was pivotal to the experiences of patients and their family-caregivers; however, it was their personable qualities, which became the central factor contributing to a higher standard of care. Honesty and patience were two traits, which were regularly cited, but it was the ability of staff to go above and beyond their standard duties of care which had the greatest impact.

One nurse last night, she came and sat with us, myself and my brother and sister and she just sat with us and explained what was happening. She was so kind and calm and gentle. Certainly my brother, who was just touched by how at the end of a long shift, she was able to just give us that time and explain how things were and they never seem to be rushed. It’s always. . . whatever we need, it’s been great (Male Family-caregiver, Inpatient unit).

Commonly, participants highlighted the value of staff availability. The increased contact time resulted in the provision of a fundamentally different service to that provided by alternative clinical settings.

They’re very caring, and you know, they always come up, would you like this would you like that. You know, they’re just so nice, and even when we play games and things, charades and bowls. They’re really nice, they join in with you, you know they don’t. . .you know like some places, you see the staff going up talking, they don’t here, they come in and they mix with you and that’s what I like (Female Patient, Day therapy).

Notably, participants often reflected upon their past experiences of care and drew comparisons between clinical settings and hospice settings. As a consequence of limited resources, high patient turnover was commonplace, which resulted in insufficient levels of contact time between patients and staff in other clinical settings which denoted a lack of care.

I have nothing but positives to say about them, it’s just changed my opinion of hospice care. They actually do care, and compared to any hospital in this Trust or even outside this Trust, it was far superior care. You felt they care. You often don’t feel that in a hospital (Male Patient, Inpatient unit).

The negative connotations associated with the term ‘hospice’, however, often acted as a barrier to early referral. Post-admittance, though, participants regularly noted the stark contrast between their original preconceptions and the reality of the hospice care they received.

People annoy me, they tend to think of, it’s a hospice, well you go there you only go there to die. That’s not true, that’s not, that’s not true by a long chalk; yes there are some people who are on their own going for end of life and there’s not a lot they can do about it, but they certainly make everybody feel prepared and ready for it and cared for. But, if there is any way they can get you up and running again they will do; they’ll move heaven and earth to do it, they really do (Male Patient, Day therapy).

Patients disclosed that preparedness for death was a valuable component of hospice care ‘*they just help me sort out a lot of things, like the do not resuscitate thing, well they sorted that out for me so that’s all done with now, so I’m just fighting for every day I can get now (Female patient, Inpatient unit)’.*

### Staff and volunteer perspectives

The perspectives of hospice personnel aligned with those of patients and family-caregivers in that they recognized the value of spending time with patients and their families. Unlike in other care settings, hospice staff were able to have more contact time, although their accounts did highlight barriers such as increased paperwork which could negatively impact their ability to maintain high levels of contact time. A reduction in contact time is likely to negatively affect a service which is regularly described as the ‘gold standard’.

Like we had two weddings last week. You know, we do our best, we have christenings, we had a horse in the other week, a lady wanted to see her horse so you know, you wouldn’t get any of that in a hospital (Hospice personnel).

## Greater support networks reduced social isolation and loneliness

### Patient and family perspectives

The diagnosis of a life-limiting illness was often considered to be an isolating experience which contributed heavily to significant psychological decline. Loneliness, depression and a lack of understanding from others were issues which featured prominently across patient interviews and, when coupled with the absence of an adequate support network, further compounded their deterioration.

Do you know, to be honest, right before I came to the hospice, I had nobody (Female Patient, Day therapy).

Notably however, in instances where a strong support network was present, often the heightened sense of isolation had not been alleviated. This was understood to be a consequence of the inability of the patient’s support network to fully comprehend all that living with a life-limiting illness entails. Subsequently, the patient’s sense of loneliness was exacerbated.

I’ve got loads of good friends, but none of them have got cancer. Bless them, you know? They really, really think the world of me, but they can frequently make me feel quite sad and worried, because they’re concerned about me having cancer, whereas these friends here, we’re all in the same boat. . . so we don’t seem to upset each other at all (Female Patient, Day therapy).

Peer support, obtained through continued attendance at a day therapy unit, provided a forum whereby patients could share their experiences. In turn, this helped to create a sense of camaraderie, thus resulting in an overall improvement in their general well-being and an alleviation of previously noted deficiencies.

You always think to yourself, well, there’s somebody worse off than me, you know? You feel sorry for yourself sometimes, and then think, well, they have a lot more to be sorry for than I have (Female Patient, Day therapy).

Families also recognized the overwhelming patient benefit that peer support facilitated, as evidenced in the following excerpt.

I think it is definitely valuable. For instance, there is two other ladies who are motor neurone disease. So, again, not that you’d ever wish this awful disease on anyone, but it was almost a comfort when she came home and said, there’s this other lady, because they’re going through and understand. It helps you put things in perspective. It’s not a competition, but at the same time, you do actually see other people there who are maybe better off, but there are people there who are worse off too (Male Family-caregiver, Day therapy).

Although patients received appropriate support, some families felt unsupported, and frequently deemed caring for a loved one as an isolating experience.

And I am a sole carer. So, I would have felt very much more isolated. And also, there are couples here as well. With this sort of thing, the change in the relationship is enormous, and you don’t realise until it’s happening how very big the changes are. It can be simple things, like, you know, [patient name] still makes the cup of tea. Well, the coffee. I’m useless. . . But, you know, it’s other things. It’s everything else you’re responsible for, and it can be pretty heavy. And that in itself can be pretty isolating (Female Family-carer, Day therapy).

Further to this, there was no evidence to suggest that families had adequate access to a social support network whereby the views and experiences of like-minded individuals could be shared. Peer-to-peer support within family units was not referred to in the accounts of family-caregivers.

### Staff and volunteer perspectives

Hospice personnel echoed the sentiments of patients and family-caregivers by reiterating the importance of peer support for patients. Staff believed that patients struggled to discuss their illness with those who had no experience of living with a life-limiting illness. This therefore exacerbated feelings of loneliness.

[T]hey want to talk about the side effects quite a lot and they all pass on little bits of knowledge – have you tried this, have you tried that and it is lovely. . .. Quite often, they will say I can’t talk to my family because their families don’t want to talk about it, they just want to talk about them getting better, but it is important for them to talk about it (Hospice personnel).

While there was no data to refute that there was an absence of formal social support measures for family-caregivers including opportunities for informal peer-to-peer support, hospice personnel recounted many examples of how they were able to provide other supportive measures.

You can sometimes go and see somebody and you’ll spend longer with their carers than you will with the patient themselves because they need that support and the same level of reassurance and care that everything is OK, that they’re doing everything that they should and it’s just reassuring them isn’t it that we will manage this and we will get through it. Even though the outcome isn’t good (Hospice personnel).

## Provision of information and advice improved patient autonomy

### Patient and family perspectives

Due to the unpredictable trajectory associated with a palliative care diagnosis, unrestricted access to support was pivotal to both the patient’s and their family’s sense of comfort, particularly in instances where specialized advice was warranted.

He’s under a lot of clinicians and you get these worrying niggles that something is happening and you think, ‘Is that . . .?’ But he comes here every Wednesday and they’ve got a doctor here. So I can pop in and say to the staff here ‘I’m concerned about this’. So it’s a complete medical back-up for me. You know that’s very important (Female Family-carer, Day therapy).

Easily accessible advice obtained through methods such as informal ‘drop-ins’ and telephone support were said to help with mood and the mitigation of worries. In many instances, this could potentially prevent unwanted hospital admissions. Across many accounts, patients reiterated that they would rather stay at home than be admitted to hospital.

For patients, palliative diagnoses carried a substantial financial burden for themselves and their families, which could diminish their quality of life. The financial burden could be dictated by factors such as household income, socio-economic status, marital status or the extent of the disease. For example, in some instances, a diagnosis resulted in the loss of employment and associated income, thus resulting in an inability to cover related expenses such as childcare, domestic help and medical equipment.

My finances were really in a mess because I had to stop work, and I hadn’t worked long enough to receive statutory sick pay, so I only had one month of statutory sick pay. Then from January to April, I didn’t receive any income at all. So when I started coming here, that was quite a priority with me, that I needed help to try and figure out what was going on, and they referred me to lots of different people, and the welfare officer came to see me. He started the ball rolling on getting me – what’s it called? ESA (employment support allowance). And that’s a long process. So that’s a really practical thing. I was trying to support two children at home with no money coming in, and so that was something – I needed to see somebody every week until that got sorted. It was just lovely the way they [hospice] made everything so easy (Female Patient, Day therapy).

### Staff and volunteer perspectives

Hospice personnel recognized that the allayment of fears was an important issue for patients that could be achieved in part through sharing and clarifying information regarding complex issues such as diagnosis and prognosis alongside information on service availability and support.

I think the majority of patients, their end would be very different, we help them to a degree to accept what’s going to happen and talk through the fears where they can’t with their family and I think they will miss out on that and I’m not saying they aren’t afraid but I think they are less afraid and more aware of their illness then they would be if we weren’t here (Hospice personnel).

## Symptom management reduced psychological distress

### Patient and family-caregiver perspectives

Despite the progressive and deteriorating nature of conditions requiring palliation, caring for patients at a hospice facility often resulted in improvements in both physical and psychosocial well-being. Due to the unpredictable trajectories typical of a palliative diagnosis and the natural deterioration associated with it, the management of symptoms was often challenging and required a flexible approach. Perhaps surprisingly, a substantial finding unearthed suicidal ideations in a proportion of participants prior to their admission.

The nurses are there for you to talk to if you need to because I have some really bad days . . . I didn’t want to be here, it was that bad. . . so, without the hospice, I don’t think I would still be here to be honest with you, because they were fantastic, and they still are. . .I’d got to the point where I just didn’t want to be here anymore. It was that bad the pain (Male Patient, Day therapy).

While psychological symptoms in patients with a terminal illness were prevalent, participants were not always explicit regarding their intent and their meaning was assumed. Subsequently, it could be deduced that prior to their admission to a hospice facility, depression amongst patients was commonplace.

I wasn’t seeing anybody, I’d just sit there and I thought, do you know what, I feel as if I’m just sitting here waiting to die (Female Patient, Day therapy).

This state of mind was somewhat reflective of the wider hospice population, although to varying degrees as the general consensus was that the absence of hospice care would have dire consequences. One patient said that they would probably:

[k]ill myself or do something to myself (Male Patient, Day therapy).

Through access to a range of services and support mechanisms, however, patients were able to adapt to their circumstances. Ultimately, they were able to establish coping methods and benefited immensely from symptom management schemes, resulting in reduced feelings of worry.

### Staff and volunteer perspectives

As the terminal nature of the disease unfolded, hospice personnel reiterated the range of psychological challenges faced by patients and family-caregivers. At a time when these two stakeholder groups are aiming to cope with concurrent losses of independence, status, a sense of self and in some cases communication, extensive psychological challenges exist. These include, but are not limited to, depression, anxiety and fear of death.

I think for people that have a terminal illness, obviously it is really difficult for them, they have a lot to take in and they get quite anxious and worried about what is going to happen and it’s almost like the illness takes over everything, it effects everything and I think that if they can have a time where they get to make music, have music therapy (Hospice personnel).

Personnel strived to minimize the impact of patient losses by helping them adapt and accept their new normal. This, in conjunction with the management of physical symptoms, provided much-needed relief to both the patients and their families.

## Improvements in patient functionality and mobility

### Patient and family-caregiver perspectives

Symptom management, whether related to the disease or a specific treatment, often had a substantial impact on a patient’s overall quality of life. The pervasiveness of symptoms for both malignant and non-malignant conditions were reported to have resulted in a high level of functional dependence. Subsequently, the inability to maintain the physical capabilities necessary to live autonomously resulted in a loss of independence, thus leading to a heavy reliance on others. Enhanced mobility provided patients with a modicum of relief from a substantial stressor.

I was restricted to what I could do. ‘Don’t do that, don’t do this’. But I’ve been finding lately, well, not lately. Say, for a while now. I’ll go in the garden and do a little bit of weeding for about half an hour. Come in and have a sit down, and a bit of a break, a cup of coffee. Go out, do a little, another half an hour, and then come in (Male Patient, Day therapy).

Through a range of support services such as physiotherapy, patients were strongly motivated to preserve their physical functioning through regular activity in a safe and controlled environment. As a result, patients experienced improvements (sometimes substantial) which helped them to regain some semblance of normality.

Through having the exercise on the exercise bike, that’s got my lungs working again which meant I was able to start taking the dogs for a walk. . . so that had an added benefit of giving me extra fitness as well. This last week, I was seeing the Rolling Stones in Manchester and that was a hell of a walk from where we parked the car. We took a couple of breaks but it was okay. So my fitness has improved coming here (Male Patient, Day therapy).

### Staff and volunteer perspectives

The preservation of function was considered vital to overall patient well-being as a decrease in mobility can have substantial impacts on a patient’s daily life. Therefore, the implementation of measures to initiate, preserve and in some instances improve, physical function helped patients reach important personal goals.

Assisting someone to stand and take a few steps so they could walk down the aisle (to get married) is a nice memory (Hospice personnel).

## Improvements in overall physical health

### Patient and family-caregiver perspectives

Debilitating issues such as pain, breathlessness, nausea and poor appetite were prevalent across the sample. Consequently, their relief was prioritized by patients and provided the primary motivation for hospice admission. Notably, clear communication, active pain assessment and access to immediate pain relief were crucial to the successful management of pain.

Well I was in a lot of pain with a pain at the top of my spine, which I was told by my GP was a trapped nerve. I see the doctor every day here and its very much on a one-to-one basis, which is a lot more than I get when you go to the hospital, the main hospital (Female Patient, Inpatient unit).

Sources of physical symptoms were broad, had multiple aetiologies and their relief was complex due to co-occurring symptoms.

When I came in I was basically a bag of bones tied up with a bit of loose string, but I weighed probably something under seven stone. I couldn’t stand because of pain in my ankles and knees, I couldn’t walk obviously and at that time the prognosis wasn’t very good and they were talking a matter of weeks (Male Patient, Inpatient unit).

The complexities of these cases highlighted the requirement to provide individualized approaches to not only pain management but also other facets of care such as dietary requirements with patients noting the value associated with access to personalized catering.

Food, if I don’t want to eat much, they watch what I’m eating, if nothing is down on the menu, they’ll do something special for me. . .. . . Special, spoilt, spoilt but they know that I have difficulty perhaps in swallowing and what they want to know that I’ve had something inside me, if I want scrambled eggs in the morning, afternoon and night, I can have them. I am spoilt, it makes you feel special (Female patient, Inpatient unit).

### Staff and volunteer perspectives

Personnel perceived improvements in physical outcomes for patients following combinations of individualized interventions.

If somebody was a bit sickly we could get them some anti-sickness, get them complementary therapies if they had, you know, for example sore shoulders, we can massage the shoulders; we offer physiotherapy so if somebody is breathless sorting that out for them, acupuncture for different symptom management as well, the physiotherapist does that, so by them coming over here, improving the quality of life, making them a little bit better (Hospice personnel).

## Respite relief contributed towards improved relationships

### Patient and family-caregiver perspectives

As a consequence of concurrent responsibilities often associated with the caregiver role, physical and psychological burden was common amongst family-caregivers. This stemmed from the lack of prioritization given to their general health and well-being, resulting in their needs often being overlooked. Consequently, family-caregivers were often forced to temporarily relinquish their caring role, which sometimes caused unwanted admissions to the hospice. In turn, this had a detrimental impact on patient and family–caregiver relationships. This was reflected in the resentment present in the tone of some patient accounts.

My husband can’t cope, basically; he has a chest infection so he’s poorly himself so the only choice we have was for me to give [give in] and therefore I have come and I am in here (Female Patient, Inpatient unit).

Because of their increasing needs, a patient was often no longer able to contribute the same level of constancy to their relationships as they were once able to. Over time, due to both the physical restrictions often associated with a palliative diagnosis and the growing need for emotional support from the family-caregiver, a power imbalance was created. Subsequently, the patient–family–caregiver dyad often had to manage unfamiliar depressive symptoms such as irritability and anger.

She’s [patient’s partner] not been very helpful in that respect. . . so we’ve never been ill. We don’t know what illnesses are about and now I have one and she’s difficult, really difficult. I’m a victim here, I’m the one who’s got cancer, she hates me because yes, I’m not getting better (Male Patient, Inpatient unit).

Due to the dynamic and non-linear trajectory associated with palliative patient populations, the complexity and scope of caregiving responsibilities are likely to expand over time. The negative impact of caregiving, however, can coexist with the positive; many caregivers revealed the benefits which could be derived from the experience. However, when family-caregivers were no longer able to assume the caregiving role, high levels of guilt were experienced.

I suppose for me, it was quite hard taking him in [to the hospice] because it’s giving up that caring role. I’d retired to take care of [patient name], so realising I couldn’t do it, was quite hard (Female Family-caregiver, Day therapy).

### Staff and volunteer perspectives

The physical and emotional exhaustion of some family-caregivers was a concern for hospice personnel. Often, family-caregivers’ exhaustion precipitated a patient’s admission into the inpatient unit for respite care. To help alleviate the pressure, support from the hospice was made available to all families.

She probably would have been very exhausted, the mental side to it as well because they were an elderly couple and sometimes the strain of it can also make them ill, and it’s when they come here, it gives the carer that day to go off and do her hair or go shopping, knowing that he is safe and cared for here. Not like when she leaves him at home and she’s rushing back because he’s on his own or he needs something (Hospice personnel).

## Discussion

Findings provide important insights into the valuable role that hospices play in end-of-life care and the positive impact they have on the quality of life of patients and their families. A palliative diagnosis has detrimental impacts on a patient’s psyche and findings show the importance of accessible support networks for patients and their family members. In particular, four patient participants reported suicidal ideations prior to their admission to the hospice. This shows the importance of early access to palliative care interventions to alleviate symptom burden, as functional impairment and pain are likely to exacerbate suicidal feelings – a finding which is replicated within the wider literature.^16^ Rumsey *et al*.^17^ reported that 60–90% of patients with advanced cancer experience excruciating pain, which contributes heavily to caregiver anxiety. However, while hospice personnel from inpatient units often prioritize the management of physical symptomology such as pain through opioids, complementary therapies such as reiki and acupuncture were often heralded by patients for their potential to reduce physical symptoms. Dingley *et al*.^18^ in their integrative review reported that a number of studies revealed statistically significant improvements in symptoms such as pain and discomfort following patient access to biofield therapies such as massage and acupuncture. Subsequently, it is important that hospice care provision continues to offer access to non-traditional forms of treatments for both patients and family-caregivers.

The isolating nature often associated with the caregiving role was clear. Caregivers, many of whom transformed from spouse to caregiver, voiced concerns regarding the insufficient support networks available to them *via* formal hospice routes. Peer support networks offer social support, social comparison and experiential knowledge which proved beneficial for patients in this study, therefore it is a priority for the future development of services that formal mechanisms for peer support should be extended to caregivers. The needs of family-caregivers however were often left unmet. The family-caregivers reported that their needs such as stress, time away and their ability to engage in their usual activities were not addressed as they nor healthcare professionals identified their needs as priority areas. Horseman *et al.*^19^ recognized that caregivers often disregard their personal needs which prevents them from accessing the support they require. Awareness of the unmet needs of family-caregivers among healthcare professionals and the facilitation of open communication can help alleviate these barriers to help-seeking. As such, ensuring family-caregiver needs continue to be identified and met must be a priority for future service development. While in this study, respite care was also found to be a critical tool to alleviate the challenges associated with caregiving, this cornerstone service remains under-researched and underutilized.^20^ Despite the potential benefits for family-caregivers and the likely prevention of unwanted and preventable hospital admissions, respite care was not always welcomed by patients in this study. Bardsley *et al.*^21^ found that on average, adults in their last year of life experienced 2.3 hospital admissions which equated to 30 bed days. Dying in hospital has been regularly cited as the least cost-effective option.^22^

Research describing the substantial pressures on the NHS is regularly cited in the wider research^23^ as well as being a theme in this study. Patients consistently referenced how the care they had received at the hospice had surpassed any care received from a hospital admission. Hospice staff were deemed to be more patient centric as they were able to afford substantially more time with patients, a finding which was not mirrored by hospice staff as they worried that the abundance of paperwork often hindered effective clinical time management. Despite the positive attributes of hospice care that were highly valued, the negative connotations associated with hospices were prevalent across patient accounts in this study as they acknowledged the stark contrast between their preconceptions of hospice care and the reality of what they experienced. Although efforts have been made to dispel such predeterminations, most notably the ‘End of Life Care Strategy’,^24^ cultural shifts are slow and negative connotations continue to act as a barrier to early referral, and the take up of referrals by patients thus highlighting this as an area requiring further development.

## Strengths and limitations

Overall, data collection was successful and, although some of the final interviews with palliative care patients were short, the information gleaned from these exchanges was invaluable. There were instances however, in which individuals were difficult to engage or, in the case of the patient with multiple sclerosis and subsequent communication impairment, challenging. If resources were available, an additional ethnographic approach including non-participant observation could have been adopted to further explore these issues over time. Notably, the balance of recruitment between day therapy and inpatients was substantially biased towards day therapy services, despite attempts being made to promote recruitment in inpatient units through regular attendance at each hospice site. Due to the trajectory of a palliative diagnosis coupled with staff gatekeeping, recruitment proved inherently difficult despite regular gatekeeper engagement. Furthermore, the transferability of findings is limited as patients with non-malignant diagnoses were largely absent from the study and there was a lack of cultural diversity. As such, the findings were not representative of the views and experiences of both minoritized communities and those with a non-malignant diagnosis. When reviewing the wider literature, it is clear that this gap in the evidence base is an issue that extends beyond Wales.^25^ Finally, as purposive sampling was used to recruit both staff and volunteers, an element of bias exists. However, as staff and volunteers were not reflecting on their own experiences, and instead were providing an insight in to patient perspectives, the bias has largely been negated. It must be recognized, however that dyadic interviews can have a number of barriers and in this research study, it is likely that both caregivers and patients minimized their experiences so as to not upset each other.

## Conclusion

This study is the largest study to explore what patients and family-caregivers value from hospice care and clearly demonstrates that hospice care provides a truly needs-led and strengths-based service, made possible by highly trained staff and volunteers. This approach is essential when supporting patients and family-caregivers who are navigating the complexities associated with a terminal diagnosis and extends beyond symptom management and pain relief, which is often viewed as synonymous with end-of-life care. Future research should explore the underutilization of hospice care by patients from minoritized communities and provide an evidence base to ensure that any institutional barriers to help seeking are removed. Furthermore, there is a need for government support to implement policies which ensure greater availability of hospice care, in part as a mechanism to reduce the burden on hospital inpatient services and to address the lack of provision of sustainable funding as this has demonstrable impacts on the availability of service provision, particularly to those with a non-malignant diagnosis who are often excluded from hospice care due to their longer life expectancies. Arguably, there are many facets of hospice care which can be outsourced to other agencies such as the administration of financial advice, however, this will inevitably have an impact on service delivery as the unique ‘one stop shop’ service provided by hospice care ensures that their provision is truly needs led.

## Supplemental Material

sj-docx-1-pcr-10.1177_26323524241231820 – Supplemental material for “Before I came to the hospice, I had nobody”. A qualitative exploration of what patients, family-caregivers, clinicians and volunteers valued most about home, day therapy or inpatient hospice services Supplemental material, sj-docx-1-pcr-10.1177_26323524241231820 for “Before I came to the hospice, I had nobody”. A qualitative exploration of what patients, family-caregivers, clinicians and volunteers valued most about home, day therapy or inpatient hospice services by Nicole Marie Hughes, Jane Noyes, Carys Stringer and Trystan Pritchard in Palliative Care and Social Practice

## Declarations

**Ethics approval and consent to participate:** This study was approved by the NHS Wales Research Ethics committee (17/WA/0399). Written informed consent to participate was obtained from participants or a consultee prior to participation.

**Consent for publication:** Informed consent for publication was provided by participants.

**Availability of data and materials:** The data is not publicly available due to privacy and ethical restrictions.
